# Associations between glucocorticoids and sociality across a continuum of vertebrate social behavior

**DOI:** 10.1002/ece3.4059

**Published:** 2018-07-02

**Authors:** Aura Raulo, Ben Dantzer

**Affiliations:** ^1^ Department of Biosciences University of Helsinki Helsinki Finland; ^2^ Zoology Department University of Oxford Oxford UK; ^3^ Department of Psychology University of Michigan Ann Arbor Michigan; ^4^ Department of Ecology and Evolutionary Biology University of Michigan Ann Arbor Michigan

**Keywords:** animal personality, cooperation, cooperative breeding, glucocorticoids, hypothalamic‐pituitary‐adrenal axis, pair‐bond, parental care, social behavior, stress

## Abstract

The causes and consequences of individual differences in animal behavior and stress physiology are increasingly studied in wild animals, yet the possibility that stress physiology underlies individual variation in social behavior has received less attention. In this review, we bring together these study areas and focus on understanding how the activity of the vertebrate neuroendocrine stress axis (HPA‐axis) may underlie individual differences in social behavior in wild animals. We first describe a continuum of vertebrate social behaviors spanning from initial social tendencies (proactive behavior) to social behavior occurring in reproductive contexts (parental care, sexual pair‐bonding) and lastly to social behavior occurring in nonreproductive contexts (nonsexual bonding, group‐level cooperation). We then perform a qualitative review of existing literature to address the correlative and causal association between measures of HPA‐axis activity (glucocorticoid levels or GCs) and each of these types of social behavior. As expected, elevated HPA‐axis activity can inhibit social behavior associated with initial social tendencies (approaching conspecifics) and reproduction. However, elevated HPA‐axis activity may also enhance more elaborate social behavior outside of reproductive contexts, such as alloparental care behavior. In addition, the effect of GCs on social behavior can depend upon the sociality of the stressor (cause of increase in GCs) and the severity of stress (extent of increase in GCs). Our review shows that the while the associations between stress responses and sociality are diverse, the role of HPA‐axis activity behind social behavior may shift toward more facilitating and less inhibiting in more social species, providing insight into how stress physiology and social systems may co‐evolve.

## 1 INTRODUCTION

Studies of individual differences in behavior have a long history in animal behavior and comparative psychology (e.g., Hall, 1934; Hall & Klein, 1942; Hinde, Bowell, & Spencer‐Booth, 1964). Contemporary research on this topic focuses on whether individuals consistently differ in their behavior across contexts (animal personality: Réale, Reader, Sol, McDougall, & Dingemanse, 2007) and whether multiple behaviors co‐vary within individuals (behavioral syndromes: Sih, Bell, & Johnson, 2004; Sih, Bell, Johnson, & Ziemba, 2004). Much of the recent research focuses on the ultimate causes of animal personality traits and behavioral syndromes as well as their possible evolutionary consequences (e.g., Dingemanse & Wolf, 2010; Réale, Dingemanse, Kazem, & Wright, 2010; Wolf, Van Doorn, & Weissing, 2008).

Empirical studies of the proximate causes of animal personality and behavioral syndromes are also increasing in frequency (Carere, Caramaschi, & Fawcett, 2010; Hau, Casagrande, Ouyang, & Baugh, 2016; Sih, 2011) and several excellent reviews highlight that the neuroendocrine stress response and animal personality or coping syndromes may co‐vary in laboratory animals (Carere et al., 2010; Koolhaas, de Boer, Coppens, & Buwalda, 2010; Koolhaas et al., 1999; Sih, 2011). The neuroendocrine stress response and the resulting changes in circulating glucocorticoid “stress hormones” may indeed play a strong modulating role in animal personalities and behavioral syndromes both through their organizational (developmental stress) and activational (acute stress) effects on behavior.

In this review, we examine how variation in the vertebrate neuroendocrine stress axis contributes to variation in social behavior. The proximate mechanisms underlying animal personalities or individual‐variation in behavior have already been reviewed to some extent (Carere et al., 2010; Hau et al., 2016; Koolhaas et al., 1999, 2010; Packard, Egan, & Ulrich‐Lai, 2016; Sapolsky, 1990; Sih, 2011). However, the predominant focus of these previous studies has been on repeatable differences in personality traits such as aggression, activity, or docility (reviewed by Bell, Hankison, & Laskowski, 2009). Although less attention has been given to social behaviors, repeatable individual differences in social behavior have also been documented (Bergmüller, Schürch, & Hamilton, 2010). Our focus is therefore distinct from these previous studies as we focus specifically on understanding the role of the neuroendocrine stress response in generating variation across a range of social behaviors.

Our overarching hypothesis is that the activity of the vertebrate neuroendocrine stress axis plays a significant role in generating individual variation in social behavior. This hypothesis is rooted in studies with laboratory animals showing that sustained increases in glucocorticoid levels (GCs) may affect social behavior by either increasing the production of corticotropin‐releasing factor (CRF) that may reduce social behavior by activating fear‐related brain circuitry (Schulkin, Morgan, & Rosen, 2005) or by shifting the effects of CRF on neural circuitry involved in reward and promoting social aversion/withdrawal (Lemos et al., 2012).

**Figure 1:**
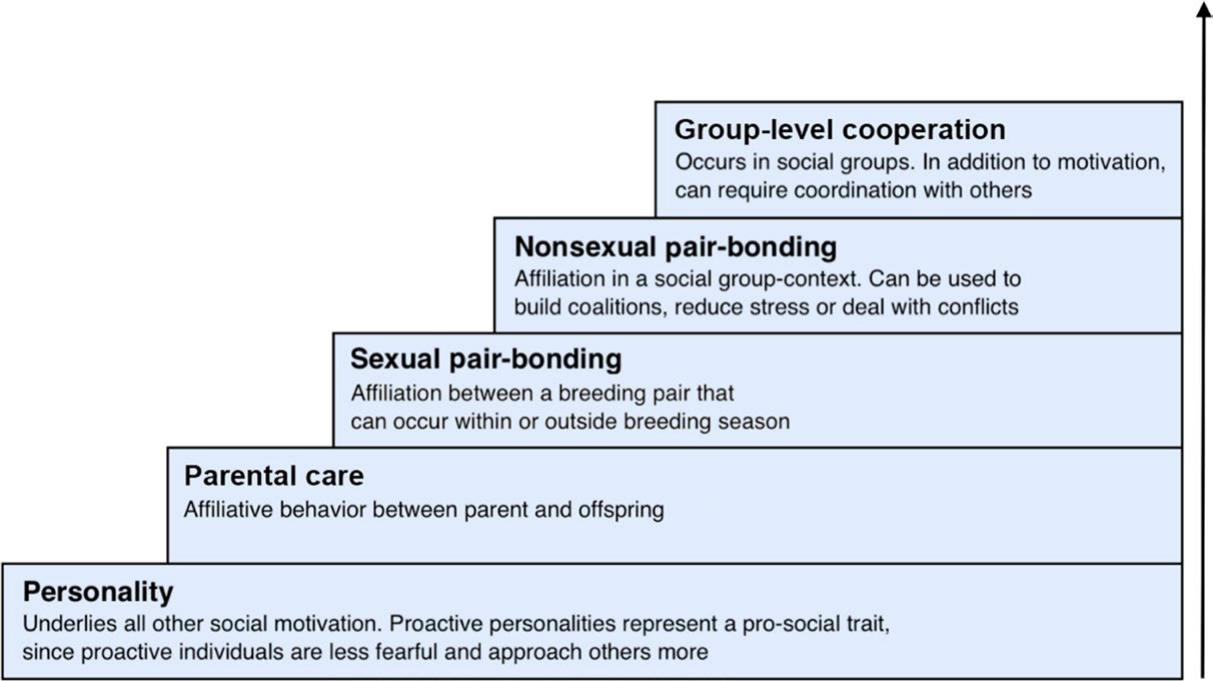
Different categories of social behavior discussed in this review. Categories range from initial social tendencies (proactive personality) to social behavior occurring in reproductive contexts (parental care, sexual pair‐bonding) and lastly to social behavior occurring in a nonreproductive contexts (nonsexual bonding, group‐level cooperation)

We first sort social behaviors into categories (Figure 1) and provide an overview of social behavior on different scales, from initial social tendencies such as how likely they are to approach a conspecific to more elaborate social behaviors apparent in highly social species (e.g., bonding behavior, parental and alloparental care). We then ask if increased neuroendocrine stress‐axis activity, such as elevated levels of glucocorticoid hormones, promotes or inhibits the expression of social behavior in each behavioral category.

We focus on research from wild vertebrate animals but inform our predictions about this research from laboratory or captive studies. The majority of the previous work on this subject has been done on laboratory or captive populations but the relationship between hormones and behavior may differ in a laboratory or captive setting versus natural habitats (Calisi & Bentley, 2009). In addition, captivity and/or captive breeding may reduce overall variation in the vertebrate neuroendocrine response (e.g., selectively eliminating individuals with a heightened or prolonged stress response: Trut, Oskina, & Kharlamova, 2009) and because captivity itself may reorganize the neuroendocrine stress axis (Dickens, Delehanty, & Romero, 2009).

## 2 DIVERSITY OF SOCIAL BEHAVIOR

Animal sociality encompasses a variety of different social behaviors. We portray this variation in social behavior as a continuum (Figure 1) by sorting different social behaviors into categories, starting from “proactive behavior” present in all species, such as boldness in approaching conspecifics or a lack of fear of conspecifics, and proceeding to more interactive behaviors, such as sexual pair‐bonding or parental care, present in more social species. Our categories build on top of each other, with lower steps representing social behavior present in a most species (e.g., reproductive behavior) whereas higher steps represent social behavior present only in some social species (e.g., cooperation). This is because many of the more elaborate social behaviors exhibited by highly social species are variations of behaviors familiar from sexual contexts in all species, but might have a very different hormonal basis. For example, while courtship and mating are social behaviors, bonding or affiliative behavior outside of an immediate mating context can perhaps be thought to be more social and less sexual. We note that our categorization of social behaviors along a continuum is conceptual and not a rigorous argument for the order in which these behaviors evolved (see Goodson, 2013).

Several of the behaviors in our categories of social behavior (Figure 1) have already been shown to exhibit repeatable individual differences where some individuals exhibit more of the given social behavior than others regardless of circumstances or context. Proactivity has been shown to be repeatable over time (reviewed by Réale et al., 2007) and there are consistent individual differences in parental behavior (Budaev, Zworykin, & Mochek, 1999; Fairbanks, 1996; Maestripieri, 1993; Schwagmeyer & Mock, 2003), affiliative behavior (Seyfarth, Silk, & Cheney, 2012; Webb, Franks, Romero, Higgins, & de Waal, 2014), and alloparental behavior in cooperative breeders (Carter, English, & Clutton‐Brock, 2014; English, Nakagawa, & Clutton‐Brock, 2010). Furthermore, individual differences in measures of HPA‐axis activity exist (Baugh, van Oers, Dingemanse, & Hau, 2014; Ellis, Jackson, & Boyce, 2006; Fletcher, Dantzer, & Boonstra, 2015) and has been observed to co‐vary with different behavioral types (Carere et al., 2010; Cockrem, 2007; Koolhaas et al., 1999; Korte, Koolhaas, Wingfield, & McEwen, 2005). In this review, we focus on the potential for these individual differences in HPA‐axis activity to cause the individual variation across the range of social behaviors (Figure 1), though we recognize that much of this work is correlative.

## 3 QUANTIFYING THE VERTEBRATE NEUROENDOCRINE STRESS RESPONSE

The vertebrate neuroendocrine response to intrinsic or extrinsic environmental challenges is initiated in the brain and results in other physiological and behavioral responses that act to maintain some homeostatic set point. These set points can vary due to a variety of intrinsic (e.g., life‐history stage) and extrinsic (e.g., season, food‐availability) factors but the main outcome is that the organism maintains some level of consistency through these times of change (i.e., allostasis: Sterling & Eyer, 1988; McEwen & Wingfield, 2003). The maintenance of allostasis is mediated in part by the neuroendocrine stress axis: the hypothalamic‐pituitary‐adrenal (HPA) ‐axis in mammals and birds or the hypothalamic‐pituitary‐inter‐renal (HPI) ‐axis in fish, amphibians, and reptiles. The most often measured product of the HPA or HPI ‐axis are the steroid hormones called glucocorticoids, which include both cortisol and corticosterone (we will refer to these collectively as glucocorticoids). The release of glucocorticoids (GCs) from the adrenals has widespread effects on physiology and behavior, which are reviewed elsewhere (Landys, Ramenofsky, & Wingfield, 2006; Sapolsky, Romero, & Munck, 2000; Wingfield & Romero, 2001).

Three different measures of GCs are commonly used to document HPA‐axis activity in wild animals. First, “*baseline GCs*” refer to GC levels under baseline or “unstressed” conditions where the individual is not experiencing any specific external challenge (baseline HPA‐axis activity). Acute stress, such as what occurs during the process of acquiring blood samples, rapidly elevates GCs (Boonstra & Singleton, 1993; Romero, Meister, Cyr, Kenagy, & Wingfield, 2008; Romero & Reed, 2005). Samples for baseline GCs are therefore commonly taken within 3 min of initial capture or restraint, though GCs might be elevated even during this time (Romero & Reed, 2005). Blood samples taken more than 3 min after initial capture do not reflect true baseline levels and are here labeled as “nominal baseline GCs” (after Delehanty & Boonstra, 2009).

Second, measures of GCs following a stressor, “*stress‐induced GCs*”, are also commonly used. Stress‐induced GCs reflect HPA‐axis reactivity and stressor intensity and they are manifold higher compared to baseline GC levels (Romero & Reed, 2005). They are sometimes measured after a standardized experimental stressor (Fletcher et al., 2015; Romero et al., 2008; Wingfield, O'Reilly, & Astheimer, 1995) or measured after a live‐trapping or handling event in the field (Delehanty & Boonstra, 2009). In addition to external challenges (e.g., being pursued by a predator) evoking variable hormonal responses, individuals may also consistently vary in their reactivity to these stressors.

The third measure commonly used in studies of stress physiology in wild animals is urinary, fecal, excreta, feather, or hair GC metabolite levels (hereafter we just add “GCs” though we recognize they are often metabolites). Urinary, fecal, and excreta GCs (Sheriff, Dantzer, Delehanty, Palme, & Boonstra, 2011) as well as feather/hair GCs (Bortolotti, Marchant, Blas, & German, 2008; Lattin, Reed, DesRochers, & Romero, 2011) are thought to reflect a mixture of both baseline and stress‐induced GC levels over a species‐specific period of time and are here called “*integrated GCs*”.

In addition to these observational methods to measure glucocorticoids, experimental studies also use different ways (e.g., hormone implants) to manipulate individual GCs (Sopinka et al., 2015). Experimental studies are extremely important given the bi‐directional relationship between hormones and behavior. We review studies that have assessed the association of social behavior and natural variation in GCs as well as when GCs were experimentally manipulated.

## 4 THE POTENTIAL ROLE OF THE STRESS AXIS IN MEDIATING SOCIAL BEHAVIOR

The role of the HPA‐axis in inhibiting or motivating social behavior is equivocal. On the one hand, it is plausible that the original role of the HPA‐axis was to *inhibit* rather than encourage social behavior. For example, the most basic form of active social behavior exhibited by many species is mating, which is mediated in part by the hypothalamic‐pituitary‐gonadal (HPG) axis. Elevated HPA‐axis activity can not only suppress the HPG axis and therefore inhibit reproductive behavior (Kirby, Geraghty, Ubuka, Bentley, & Kaufer, 2009) but it can also trigger abandonment of offspring (Wingfield et al., 1998). Consequently, increased activity of the HPA‐axis can have an inhibitory role on these basic social behaviors (tolerance, mating, and parental care).

On the other hand, the effects of GCs on social behavior may depend on the social and environmental context (Orchinik, 1998) as well stressor severity (Lemos et al., 2012). GCs are regarded mainly as permissive or facilitating hormones behind many behaviors, such as self‐grooming or feeding (Katz & Roth, 1979; Packard et al., 2016; Rowland & Antelman, 1976). Because many of these behaviors also occur in a social context (e.g., allogrooming, feeding offspring, or hunting in a group), an elevation of GCs may not always inhibit social behavior. For example, GCs may promote as well as inhibit aggression toward conspecifics depending on social rank, personality and the duration of the stressor (Grace & Anderson, 2014; Summers et al., 2005). Furthermore, while acute HPA‐axis activity may trigger dopamine production, severe stress is known to abolish this rewarding effect and lead to social withdrawal (Lemos et al., 2012).

**Figure 2:**
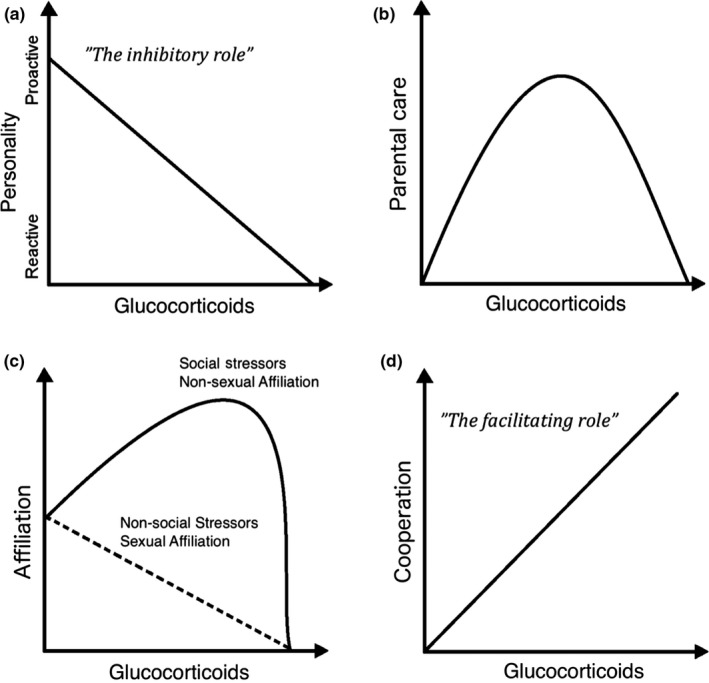
A predictive framework for the effects of glucocorticoids (GCs) on different categories of social behavior. (a) At the level of initial social tendencies to approach conspecifics (proactive personality), increasing GCs are expected to be inversely associated with social behavior (“*The inhibitory role of GCs*”). (b) In the context of parental care, GCs may affect this form of social behavior in a nonlinear fashion, facilitating it in moderate levels but inhibiting it at very low or very high levels. (c) In the context of pair‐bonding or affiliative behavior, GCs may inhibit social behavior in sexual contexts but facilitate it in nonsexual contexts (see Figure 1 for description of these two types of affiliation). The latter trend is likely limited to a threshold level of GCs beyond which GCs no longer promote social behavior. Furthermore, solely nonsocial environmental stressors are likely to have more negative effect on affiliation than social stressors. (d) In the context of group‐level cooperation, GCs increase social behavior without a threshold level (“*The facilitating role of GCs*”)

These studies suggest that the effects of GCs on social behavior may be more nuanced such as being affected by the sociality of the species or the type and severity of the stressor. We performed a literature search for studies that had examined the association between GCs and any of the social behaviors in Figure 1 in wild animals. As in other studies, we assumed that individuals with elevated GCs (baseline, stress‐induced or integrated) corresponded to individuals with higher overall HPA‐axis activity. We then qualitatively assessed patterns of covariation between GCs and social behavior. We predicted that across our categories of social behavior (Figure 1), the role of the neuroendocrine stress response shifts from inhibiting (“The inhibitory role”, Figure 2a) to facilitating social behaviors (“The facilitating role”, Figure 2d) when the behavioral context is more social and less sexual. Within this framework and when relevant, we also examined if the association between HPA‐axis activity and social behavior depended upon (i) the sociality of the species, (ii) the severity of the stressor, and (iii) the sociality of the stressor (e.g., a nonsocial environmental stressor such as predation risk versus a social stressor of conspecific conflict: Figure 2b,c).

We expected that the effect of GCs on social behavior would follow the inhibitory role in nonsocial species whereas both the inhibitory and facilitating role of GCs would occur in more social species. Specifically, we predicted that GCs may inhibit initial social tendencies and reproductive social behavior, but that GCs would motivate social behavior in the context of social stressors and more elaborate social behaviors (shown in Figure 1).

## 5 EVIDENCE THAT VARIATION IN STRESS PHYSIOLOGY IS ASSOCIATED WITH VARIATION IN SOCIAL BEHAVIOR

### 5.1 Association between GCs and proactive behavior

We first examined if proactivity was associated with HPA‐axis activity and whether this association depended upon the sociality of the species. Proactive behavior may reflect initial motivation for engaging in social behavior where more proactive (bold, explorative, inquisitive) individuals tend to be more sociable than those that are more reactive (shy, fearful, neophobic). For example, exploration and boldness, both of which are measures of proactivity, are linked to group shoaling tendency in fish (Cote, Fogarty, Weinersmith, Brodin, & Sih, 2010; Muraco, Aspbury, & Gabor, 2014; Smith & Blumstein, 2010), social communication in social rodents (Crino, Larkin, & Phelps, 2010), social information use (Marchetti & Drent, 2000), and overall cooperativeness in some highly social birds (Scheid & Noe, 2010) and mammals (English et al., 2010). However, proactivity can also be linked to territorial aggression (Sih, Bell, & Johnson, 2004; Sih, Bell, Johnson, & Ziemba, 2004; Yewers, Pryke, & Stuart‐Fox, 2016; see also Hall, Parson, Riebel, & Mulder, 2016; Taylor & Lattanzio, 2016) and thus the association between GCs and social proactivity may depend on species sociality (e.g., whether a species exhibits territory defense, tolerance or cooperative territory defense).

#### 5.1.1 Evidence from captive animal studies

Studies from captive animals show that proactive or bold individuals tend to have lower stress‐induced GCs and that GCs may have an inhibitory role on proactive behavior. For example, Great Tits (*Parus major*) that were artificially selected for proactive personality, showed markedly lower integrated (Stöwe, Rosivall, Drent, & Möstl, 2010) and stress‐induced GCs (Baugh et al., 2012). Similarly, Japanese Quail (*Coturnix japonica*) artificially selected for lower HPA‐axis reactivity exhibited a more proactive personality (Jones, Satterlee, & Ryder, 1994). Similar results of high HPA‐axis reactivity correlating with a less proactive personality come from studies of captive house mice (*Mus musculus*) artificially selected for higher aggressiveness (Veenema, Meijer, de Kloet, Koolhaas, & Bohus, 2003), Zebra Finch (*Taeniopygia guttata*) selected for more exploratory behavior, (Martins, Roberts, Giblin, Huxham, & Evans, 2007), sheep (*Ovis aries*) selected for HPA‐axis reactivity (Lee et al., 2014), rainbow trout (*Oncorhynchus mykiss*) selected for elevated HPI axis reactivity (Overli, Pottinger, Carrick, Overli, & Winberg, 2002), and zebra fish (*Danio rerio*) selected for boldness (Oswald, Drew, Racine, Murdoch, & Robison, 2012). Finally, studies of laboratory rodents show that application of experimental stressors can increase anxiety‐like and reactive behavior (Delgado‐Morales et al., 2012; Egan et al., 2009).

#### 5.1.2 Evidence from wild animal studies

**Figure 3:**
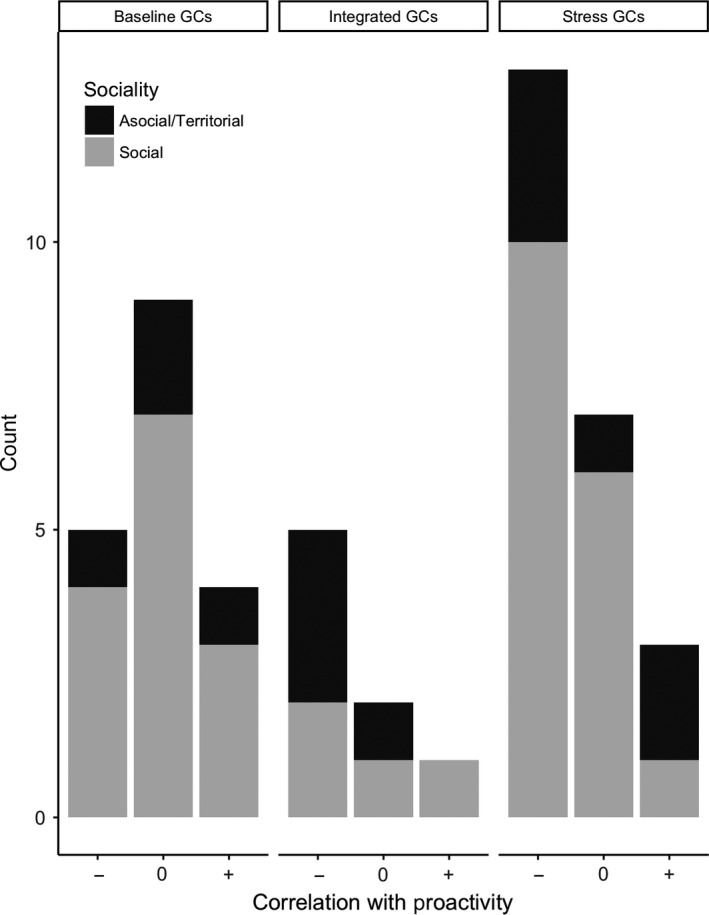
Summary of evidence between measures of glucocorticoids (GCs) and proactivity in wild vertebrates. Studies measuring stress‐induced or integrated GCs show proactivity is mostly negatively correlated with GCs, whereas studies measuring baseline GCs show mostly no correlation. Sociality of the species is not associated with the relationship between GCs and proactivity. Counts are comparisons where any measure of GCs and proactivity were investigated and in some cases there was more than one comparison per study (see Table S1)

Forty six percent (46%) of cases where proactivity and GCs were compared (Table S1, Figure 2) showed that individuals with more proactive personalities had lower GCs (Table S1, Figure 3). This trend emerged from 49 comparisons of the association between proactivity and GCs from 32 studies of 23 vertebrate species. The trend was largely driven by studies showing that stress‐induced or integrated GCs were negatively correlated with proactivity. Specifically, 56% of studies measuring stress‐induced GCs and 63% of studies measuring integrated GCs found that individuals with higher GCs were less proactive. Conversely, of the studies where baseline GCs were measured, 27% found a negative correlation, 50% found no correlation, and 22% found a positive correlation. Eight of the 32 studies investigated if the relationship between GCs and proactivity was sex‐specific and none found evidence for a sex‐specific relationship.

We found little support for the prediction that the association between HPA‐axis activity and proactivity was dependent on the sociality of the species (Figure 3). In social species, such as the colonially‐breeding common eider (*Somateria mollissima*: Seltmann et al., 2012; Seltmann, Jaatinen, Steele, & Ost, 2014), individuals with elevated GCs (baseline, stress‐induced, or integrated) exhibited less proactive behavior in 45% of studies and proactivity and GCs were positively associated in 14% of cases studies. Similarly, in nonsocial or territorial species, such as eastern chipmunks (*Tamias striatus*: Montiglio, Garant, Pelletier, & Réale, 2012), individuals with elevated GCs (baseline, stress‐induced, or integrated) were less proactive in 50% of cases studies while 21% of studies found a positive correlation between GCs and proactivity (Figure 3).

#### 5.1.3 Discussion and future directions

These results from studies of wild animals match those from previous studies in captive animals, suggesting that this measure of social behavior (proactivity) is negatively associated with HPA‐axis activity and that species sociality did not modulate the relationship between HPA‐axis activity and social behavior. Baseline GCs rarely associated with proactivity whereas studies measuring stress‐induced GCs or fecal/feather GCs found a negative association with proactivity. Because these studies were correlational we cannot readily present hormones as a cause of these behaviors and we found no studies testing for the effect of experimental stress on proactive behavior.

In contrast with studies in captive animals, a few (16%) comparisons in wild animals also found a positive correlation where proactive individuals had higher GCs. Most of the studies (4 of 6) were done with wild animals brought into captivity or were from semi‐natural populations (Adams, Farnworth, Rickett, Parker, & Cockrem, 2011; Kralj‐Fiser, Weiss, & Kotrschal, 2010; Muraco et al., 2014; Overli et al., 2007). These conditions might induce different effects on proactive and reactive individuals; reactive, behaviorally passive, individuals are thought to cope better with novel situations (Benus, Bohus, Koolhaas, & Vanoortmerssen, 1991; Cockrem, 2007; Coppens, de Boer, & Koolhaas, 2010; Ruis et al., 2001). Taking individuals from their natural habitat and bringing them into captivity could cause proactive individuals to be more stressed or responsive to stress in the novel conditions, while having little or no effect on reactive individuals. Thus, the relationship between HPA‐axis activity and proactivity may be influenced by the study design.

We found no support that the association between HPA‐axis activity and proactivity was dependent on the sociality of the species. However, not all proactive behavior (e.g., territorial aggression) is unequivocally sociable behavior and in highly social species, social behavior often has components of both proactivity and reactivity. While reactive individuals are always less sociable, proactive behavior may divide into two types: In less social species, proactive personalities can be sociable (e.g., curious, bold, approaching conspecifics of opposite sex) or aggressive (e.g., territorial behavior; see Rödel, Monclus, & von Holst, 2006; Grace & Anderson, 2014). In highly social species, proactive personalities can divide into either unresponsive to social stress (callous) or responding to social stress with sociable behavior (see Seyfarth et al., 2012).

### 5.2 Association between GCs and parental care

Parental care is a social behavior that occurs in a reproductive context. As we summarize below, the negative effects of GCs on general reproductive behavior have been widely studied, but many of the individual behaviors related to parenting (such as foraging) are also known to be enhanced by GCs. We examined the evidence of whether parental care is positively or negatively associated with HPA‐axis activity and whether this relationship depends on the magnitude of the stress response.

#### 5.2.1 Evidence from captive animal studies

Maternal care has been studied more so than paternal care, but hormonal correlates of parental care are relatively similar for both parents in species with biparental care (Harris, de Jong, Yang, & Saltzman, 2013; Miller, Vleck, & Otis, 2009; Ouyang, Muturi, Quetting, & Hau, 2013; Storey, Walsh, Quinton, & Wynne‐Edwards, 2000). In general, increased GCs have negative effects on breeding and parental behavior. For example, stress‐induced GCs can trigger nest abandonment in birds (Wingfield et al., 1998) and can impair maternal care in mammals (Jarvis et al., 2006; Brummelte & Galea, 2010; but see also Saltzman & Maestripieri, 2011). At the same time, moderate increases in GCs are typically observed during the breeding season in wild animals when they may be exhibiting parental care (Fletcher et al., 2015; Jeffrey, Cooke, & Gilmour, 2014; O'Connor, Yick, Gilmour, Van Der Kraak, & Cooke, 2011; Romero, 2002; Wingfield & Sapolsky, 2003). Laboratory studies suggest that these seasonal increases in GCs could enhance parental behavior (Bales, Kramer, Lewis‐Reese, & Carter, 2005; Fleming, Steiner, & Corter, 1997; Harris et al., 2013; Saltzman & Maestripieri, 2011). For example, male California mice (*Peromyscus californicus*) showed more paternal behavior after mild separation stress whereas chronic variable stress impaired their care behavior (Harris et al., 2013). Similarly, in primates, moderate GCs during gestation and after parturition are positively correlated with maternal behavior across mothers (Fleming, Corter, & Steiner, 1995; Fleming et al., 1997; Stallings, Fleming, Corter, Worthman, & Steiner, 2001; Bardi, French, Ramirez, & Brent, 2004; see also Storey et al., 2000). Moreover, just as GCs are known to increase self‐feeding behavior (Rowland & Antelman, 1976), they also increase feeding of young (Astheimer, Buttemer, & Wingfield, 1992; Silverin,^1986^).

Based upon this evidence, we predicted that parental care is associated with HPA‐axis activity in a nonlinear fashion where individuals with moderately high GC levels (within their species‐typical range) exhibit the highest amount of parental care. Small increases in GCs should act as a motivational signal to increase parental care while extreme increases in GCs should lead to abandonment of young when potential future reproduction exists.

#### 5.2.2 Evidence from wild animal studies

We located 37 studies of 23 vertebrate species that investigated the associations between GC levels and parental care behavior in wild animals (Table S2). Most of the studies were conducted in birds and fish and there is a noteworthy gap of studies investigating associations between parental behavior and HPA‐axis activity in wild mammals (but see Nguyen, Gesquiere, Wango, Alberts, & Altmann, 2008).

**Figure 4:**
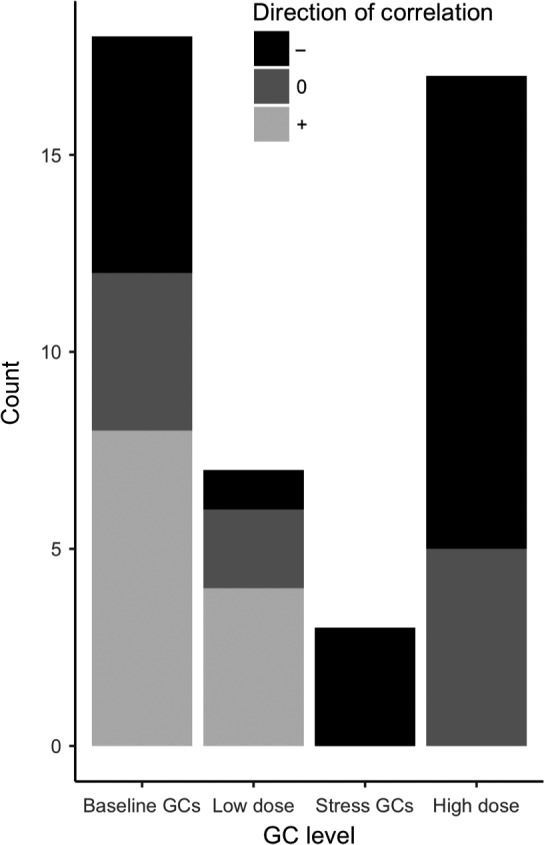
Summary of evidence on the associations between measures of glucocorticoids (GCs) and parental care in wild vertebrates. Most comparisons showed that parental care is negatively associated with GCs when they are elevated within the stress induced range or given a high dose of experimental GCs whereas positive correlations are apparent in comparisons with GCs on lower baseline range (baseline GCs or low dose experimental GCs). Counts are comparisons where any measure of GCs and parental care were investigated and in some cases there was more than one comparison per study (see Table S2)

The majority of studies supported our prediction that the relationship between HPA‐axis activity and parental care was nonlinear where slight increases in GCs elevated parental care but substantial increases in GCs decreased it (Figure 2b). Most comparisons (60%) found that individuals with high baseline GCs or low experimental increases in GCs exhibited increased parental care, whereas individuals with high stress GCs or high experimental increases in GCs exhibited decreased parental care (Table S2; Figure 4). For example, Great Tits with higher baseline GCs fed their young more (Ouyang, Muturi, et al., 2013) and baseline GCs in parents were positively associated with offspring growth (a proxy of parental care) in Mourning Doves (*Zenaida macroura*, Miller et al., 2009), female Tree Swallows (*Tachycineta bicolor*, Bonier, Moore, Martin, & Robertson, 2009) and House Sparrows (*Parus major,* Ouyang, Sharp, Dawson, Quetting, & Hau, 2011). This positive trend was found by 48% of comparisons where low increases in GCs (increases within baseline range) were measured. In contrast, when looking at high increases in GCs (within stress‐induced or supra‐physiological range), no comparison showed a positive trend and 75% found a negative trend between GCs and parental care (Figure 4). For example, House Sparrows with higher increases in GCs following a standardized stressor also fed their nestlings less (Ouyang et al., 2011).

This nonlinear relationship between HPA‐axis activity and parental care is highlighted by the studies of Silverin (1982, 1986). Silverin implanted nesting Pied Flycatchers (*Ficedula hypoleuca*) with different dosages of corticosterone. Birds with slight experimental increases in GCs exhibited enhanced parental food provisioning to offspring but higher GC increases reduced feeding of young and even higher GC increases led to nest and territory abandonment. Studies in fish species that exhibit parental care also show that the probability of nest‐abandonment or egg cannibalism became less likely with low increases in GCs but increased under high increases in GCs (Neff & Knapp, 2009; O'Connor, Gilmour, Arlinghaus, Van Der Kraak, & Cooke, 2009).

Twenty four percent (24%) of comparisons found no correlation between GCs (all three measures) and parental care and 6 comparisons (13%) found evidence contradictory to our prediction where baseline GCs were negatively correlated with parental care. Interestingly, all of these studies were done with birds and used the probability of nest‐abandonment as the proxy of parental care (i.e., lower parental care occurred when the nest was abandoned). In contrast with other measures of parental care (e.g., food provisioning), nest‐abandonment probability seems to increase linearly with increasing HPA‐axisactivity in birds. For example, even though higher baseline GCs increased chick‐feeding rate in Great Tits, higher baseline GCs also increased the probability that the nest was abandoned (Ouyang, Quetting, & Hau, 2012). This was unlike studies in fish, where nest‐abandonment probability depended on the level of GCs in the same nonlinear way as other care behaviors (Neff & Knapp, 2009; O'Connor et al., 2009).

#### 5.2.3 Discussion and future directions

Studies from both the laboratory and natural populations show that small increases in GCs increase parental behavior but large increase in GCs can impair parental behavior (Figure 2b). In addition to correlational evidence, there was causal evidence suggesting that slight increases in GCs elevate parental care. An exception to the overall trend occurs in the probability to abandon nests containing offspring in birds, which appears to be increased by even slight increases in GCs within the baseline range (Silverin, 1986; Groscolas & Robin, 2001; Love, Breuner, Vezina, & Williams, 2004; Groscolas, Lacroix, & Robin, 2008; Spée et al., 2010, 2011; Ouyang et al., 2012; Strasser & Heath, 2013; but see Criscuolo et al., 2005), perhaps because chronic elevations in baseline GCs might trigger individuals to switch to an emergency life‐history stage (Wingfield et al., 1998).

Unsurprisingly, the effect GCs have on parental behaviour depend on the timing and severity of the stressor causing the increase in GCs. The threshold where GCs start to reduce parental behavior may depend upon the life history of the species and past reproductive investment (Bokony et al., 2009; Breuner, 2011). For instance, a higher magnitude of increase in baseline GCs is needed to abandon chicks than eggs in multiple bird species (Bonier, Moore, & Robertson, 2011; Groscolas et al., 2008; Love et al., 2004). This calls for more work on understanding whether there are species‐specific thresholds whereupon elevated GCs decreases parental care. More research on the effect of timing and duration of increases in GCs is also needed to understand if their effects on parental care differ. For example, Ouyang et al. (2011), Ouyang, Sharp, Quetting, and Hau (2013) found that parental effort in House Sparrows (2011) and reproductive success in Great Tits (2013a) was positively correlated with baseline GCs during breeding but negatively correlated with pre‐breeding baseline GCs and overall HPA‐axis reactivity. These results indicate a need for more studies measuring the association between parental care and baseline GCs quantified at different life history stages (see Love, Madliger, Bourgeon, Semeniuk, & Williams, 2014; Ouyang et al., 2011).

It is of course likely that the effect of GCs on parental care, and social behavior in general, involves interplay with other hormone levels. There was evidence that the effects of GCs on measures of parental care (offspring abandonment) are contingent upon simultaneously low levels of prolactin (Groscolas & Robin, 2001; Groscolas et al., 2008; Spée et al., 2010, 2011). For instance, high baseline GCs only resulted in nest abandonment in Adelie Penguins (*Pygoscelis adeliae*) when coupled with decreased prolactin levels (Spée et al., 2010). Prolactin could maintain the motivational effects of baseline GCs on parental behavior while at the same time reducing the probability that the individual enters an emergency life history stage (i.e., abandoning a nest). The interaction between GCs and prolactin calls for more research since it may be a functional mechanism allowing the shift from the inhibitory to the facilitating role in how GCs affect social behavior.

### 5.3 Association between GCs and Affiliative Behavior

Pair‐bonding behaviors are usually defined as any type of affiliative behavior, such as grooming, huddling, preening, courting or courtship/mating behaviors between two conspecifics. These affiliative behaviors are thought to strengthen the pair‐bond between two opposite‐sex adult individuals in socially monogamous species (Bales et al., 2005; DeVries, Taymans, & Carter, 1997; Sachser, Durschlag, & Hirzel, 1998). We will refer to behaviors between two opposite‐sex conspecifics as “sexual pair‐bonding”.

Pair‐bonding behavior between two opposite‐sex individuals can also occur in the nonbreeding season and even in nonreproductive contexts, such as between two same‐sex individuals where similar behaviors (indicated above) are used to strengthen nonsexual social bonds. For example, in social species, affiliative behaviors occur between members of the same social group, independent of breeding season or reproductive partnership. We will refer to this type of affiliative behavior in a nonreproductive context as “nonsexual pair‐bonding”.

Because social and sexual relationships mirror very different attributes of individual fitness, the association of GCs and affiliative pair‐bonding behavior might depend on sexual/nonsexual context. Furthermore, the type of a stressor (environmental or social) may have differential effects on the association between GCs and bonding behavior. For example, nonsexual pair‐bonding behavior may be used to cope with social stressors through consolation and social support whereas environmental stressors (e.g., food shortage, increased predation) might trigger more nonsocial responses and thus reduce affiliation. We therefore examined the evidence of whether sexual or nonsexual pair‐bonding behavior is enhanced or reduced by GCs and whether this association depends on the context or the social nature of the stressor (i.e., whether a feature of the physical or social environment elicited the stress response).

#### 5.3.1 GCs and sexual pair‐bonding–Evidence from captive animal studies

Our literature analysis revealed that associations of stress‐induced GCs and sexual pair‐bonding behavior have only been studied in captivity. As expected, the expression of sexual pair‐bonding behavior in mating context can be reduced by elevated GCs. For example, female Song Sparrows displayed less courtship behavior when injected with corticotropin‐releasing factor (CRF), a neuropeptide that increases production of GCs (Maney & Wingfield, 1998). In contrast, outside of an immediate mating context, the formation and maintenance of sexual pair‐bonds appears to be mostly enhanced by GCs. This evidence comes mainly from social isolation or partner‐loss studies in socially monogamous species where individuals are separated from their partner or natal group, which increases in GCs, and then reunited or introduced to a new potential mate. For example, male Zebra Finches treated with exogenous corticosterone increased pair‐bond formation behaviors with novel females (LaPlante, Huremovic, & Tomaszycki, 2014). Paralleling this, female Zebra Finches with higher stress‐induced GCs (caused by isolation from partner) exhibited more pair‐maintenance behavior (affiliative proximity) to their partner after reunion (Prior & Soma, 2015). Unlike when introduced to a novel female, male Zebra finch affiliative behavior upon reunion with partner rapidly reduced GC levels back to baseline (Remage‐Healey, Adkins‐Regan, & Romero, 2003). Similarly, in male guinea pigs (*Galea monasteriensis*), increased GCs caused by partner separation triggered socio‐sexual affiliative behavior upon reunion and this was associated with subsequently reduced GC levels (Adrian et al., 2008). These studies highlight the general point that affiliative behavior (in these cases occurring after reunion of pair‐bonded individuals) can downregulate GCs in social species (DeVries, 2002; Sachser et al., 1998).

Elevations in HPA‐axis activity may also play a role in the formation of new pair‐bonds upon dispersal from the natal group. Dispersal out of a natal group is coupled with social isolation, elevating both urinary and perhaps baseline GCs (Smith, Birnie, & French, 2011; Smith, Powning, et al., 2011; Smith & French, 1997), which may promote pair‐bonding when mates are finally located. For example, in white‐faced marmosets (*Callithrix geoffroy*), individuals that had experienced social isolation before pairing (and consequently had higher baseline GCs), approached each other more frequently and spent more time in proximity with each other than those that did not experience social isolation (Smith, Birnie, et al., 2011; Smith, Powning, et al., 2011). In species with sex‐biased natal dispersal, elevated GCs may promote pair‐bonding but only in the dispersing sex. For instance, experimentally increasing GCs reduced the time required to form a pair‐bond in male but not female prairie voles (*Microtus ochrogaster*: DeVries, Devries, Taymans, & Carter, 1995; DeVries, DeVries, Taymans, & Carter, 1996; see also LaPlante et al., 2014). This reflects the social system of this species where male prairie voles wander around and disperse more so than females (Carter, 1998; Getz, McGuire, Hofmann, Pizzuto, & Frase, 1994; McGuire, Getz, Hofmann, Pizzuto, & Frase, 1993; Solomon & Jacquot, 2002).

#### 5.3.2 GCs and nonsexual pair‐bonding–Evidence from captive animal studies

Studies of captive group‐living species show nonsexual pair‐bonding behavior between group members can also be enhanced by elevations in HPA‐axis activity. For instance, higher integrated GCs were positively associated with more affiliative preening in ravens (Stöwe et al., 2008). Nonsexual affiliative behavior tends to occur after stressors and may subsequently down‐regulate HPA‐axis activity. For example, crowding is a social stressor that is associated with increased integrated GCs as well as social grooming rates in primates (De Waal, Aureli, & Judge, 2000) and social grooming is known to reduce GCs in both the groomer and the receiver (Gust, Gordon, Hambright, & Wilson, 1993). Affiliative pair‐bonding behaviors appear to have a stress‐reducing effect in general. For example, tactile stimulation can attenuate human infant's reactivity to stress (Feldman, Singer, & Zagoory, 2010; see also Soares, Oliveira, Ros, Grutter, & Bshary, 2011 for an example in fish) and reciprocal affiliative behavior is associated with lower stress‐induced GCs in rats (Yee, Cavigelli, Delgado, & McClintock, 2008).

Factors of the social environment that increase HPA‐axis activity (e.g., social conflict) can promote nonsexual pair‐bonding behavior. For example, bonobos (*Pan paniscus*) engage in socio‐sexual affiliation behaviors under the stress of aggressive conflicts between group members (Hohmann, Mundry, & Deschner, 2009; Vasey, 1995). Affiliative behavior after conflicts tends to occur between victims of conflict and other conspecifics (consolation) or between opponents (reconciliation). This bonding behavior can strengthen the social bonds between participants (Clay & de Waal, 2015; Cordoni & Palagi, 2008; De Waal & Vanroosmalen, 1979; Fraser & Bugnyar, 2011; Seed, Clayton, & Emery, 2007; Wittig & Boesch, 2010), protect from further aggression (Call, Aureli, & De Waal, 2002; Koski & Sterck, 2009; Palagi, Chiarugi, & Cordoni, 2008), and reduce baseline GCs or behavioral signs of stress in the victim (Castles & Whiten, 1998; Duboscq, Agil, Engelhardt, & Thierry, 2014; Fraser & Aureli, 2008; Fraser, Stahl, & Aureli, 2008; McFarland & Majolo, 2012).

The above evidence suggests that the social environment (e.g., conflict) can both increase HPA‐axis activity and promote nonsexual pair‐bonding behavior. However, nonsocial environmental stressors (e.g. exposure to predators or extreme weather) may not promote affiliative behavior. For instance, studies of zoo‐housed mammals report higher fecal/urine GCs and stress behavior coupled with a reduced rate of social behaviours with an increasing number of zoo visitors (Barbosa & Mota, 2009; Davis, Schaffner, & Smith, 2005; Mallapur, Sinha, & Waran, 2005; Scott, Heistermann, Cant, & Vitikainen, 2017). Furthermore, common marmosets (*Callithrix jacchus*) had higher integrated GCs and less affiliative behaviors during weekdays compared to weekends when the zoo was closed (Barbosa & Mota, 2009). This suggests the possibility that environmental stressors may reduce nonsexual pair‐bonding behavior. Given the observational nature of these studies, experimental studies to investigate these relationships are greatly needed.

#### 5.3.3 GCs and sexual and nonsexual pair‐bonding behavior–Evidence from wild‐animal studies

**Figure 5:**
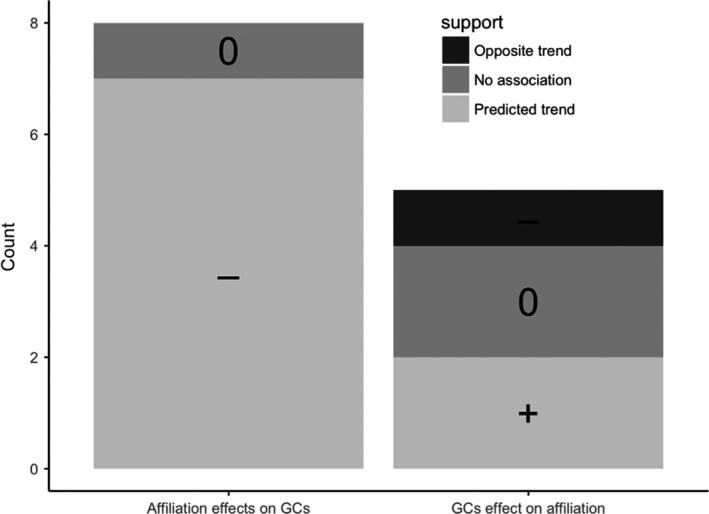
Summary of evidence between measures of glucocorticoids (GCs) and nonsexual pair‐bonding behavior in wild vertebrates. Most studies support our prediction that GCs have a positive effect on pair‐bonding behavior (affiliation) where increased affiliative behavior between two conspecific is associated with a subsequent reduction in GCs (*Affiliation effects on GCs*). Some studies also supported the prediction that elevations in GCs caused an increase in the expression of nonsexual pair‐bonding behavior (*GCs effect on affiliation*). Counts are comparisons where any measure of GCs and nonsexual bonding behavior were investigated and in some cases there was more than one comparison per study (see Table S3)

We located no studies of the relationship of sexual pair‐bonding behavior and HPA‐axis activity in wild animals, but we did find 13 studies of 9 species that had studied the relationship between nonsexual pair‐bonding behavior and GCs in wild animals (Table S3, Figure 5). All studies except one were done in primates, mostly in cercopithecine primates (baboons) that live in large social groups.

Seventy percent (70%) of the comparisons in the studies we located were consistent with our prediction, derived from captive animal studies, that elevated HPA‐axis activity would increase nonsexual pair‐bonding behavior (*GCs effect on affiliation*: Figure 5) and this behavior would in turn down‐regulate GCs (*affiliation effects on GCs*: Figure 5). This is best illustrated by Engh et al. (2006) who showed that female chacma baboons (*Papio ursinus*) that had higher integrated GCs due to a death of close relative increased their rate of grooming others and the more they groomed the lower integrated GCs they had a month after the stressful incident. The only nonprimate study was also the only one that had an experimental approach. This study showed that Adele penguins treated with high dose GCs (within the stress‐induced range) subsequently increased their affiliative (nonsexual pair‐bonding) behavior (Thierry, Brajon, Spée, & Raclot, 2014). However, instability in primate social groups (e.g., unstable dominance hierarchy) that is known to elevate GCs in group members (Creel, Dantzer, Goymann, & Rubenstein, 2013) did not affect the amount of nonsexual bonding behavior but was associated with same amount of bonding behavior (grooming) being focused to fewer group recipients (Crockford, Wittig, Whitten, Seyfarth, & Cheney, 2008; Wittig et al., 2008).

We found only one example of how environmental (nonsocial) stressors affected nonsexual pair‐bonding behaviors in wild animals (Table S3). When food was scarce, red‐bellied lemurs (*Eulemur rubriventer*) had higher integrated GCs (Tecot, 2013), reduced grooming rates and overall lower social proximity (Overdorff & Tecot, 2006) within their small family groups.

#### 5.3.4 Discussion and future directions

Evidence of the role of HPA‐axis activity behind pair‐bonding behavior in wild animals is limited to studies in nonsexual contexts but generally fits the patterns observed in captivity. Increased GCs were associated with *subsequent* increased affiliative behavior (*GCs effect on affiliation*: Figure 5) and this result is corroborated by the one experimental study that showed that affiliative behavior, unlike parental behavior, was enhanced by high dose of exogenous corticosterone in Adele penguins (Thierry et al., 2014). Contrary to our expectations, this positive effect of (even high) GCs on bonding behavior was largely independent of sexual and nonsexual context: only bonding behavior in an immediate mating context (such as courtship) was shown to be inhibited by GCs.

There was limited evidence to differentiate between the effects of elevated GCs caused by social versus environmental stressors on social behavior. Social stressors (death of close relative) increased grooming in baboons (Engh et al., 2006) whereas another type of social stressor in other species (group instability) that likely increases GCs in group members (Creel et al., 2013) did not elevate grooming among group members. In contrast, reduced food availability (a nonsocial environmental stressor) that increased GCs in red‐bellied lemurs was associated with a reduction in grooming (Tecot, 2013; Overdorff and Tecot, 2006). Whether or not the cause (environmental or social factor) of increase in GCs affects the outcome on pair‐bonding behavior is an area that is ripe for exploration. We expect that nonsocial environmental factors that elevate GCs in group members will reduce affiliative behavior whereas increases in GCs caused by social factors will increase it (Figure 2c). Affiliative grooming is unlikely to reconcile the consequences of an environmental stressor (e.g., severe snow storm or a drought), whereas it may provide tools for social support, relationship repair and aggression avoidance in face of social conflicts. However, the trend of social stressors facilitating and environmental stressors inhibiting affiliative behavior might vanish if affiliative behaviors among group members markedly improve group‐level cooperative behaviors (Smith, Birnie, et al., 2011; Bailey, Myatt, & Wilson, 2013). For example, dominant Green Woodhoopoes (*Phoeniculus purpureus*) increase preening of subordinate group members when entering areas of potential intergroup conflict, which may help “to get the soldiers in line” (Radford, 2011). When environmental or inter‐group stressors can be fought with numbers, GCs may increase social bonding to promote cooperation to fight the stressor, regardless of its nature.

### 5.4 Association of GCs and group‐level cooperation

Group‐level cooperation within a social group is here defined as coordinated target‐based social action between more than two individuals that can have mutual benefits for group members. Group‐level cooperation often consists of the same behaviors that are performed by less social species (e.g., parental care, hunting) but in a social context and often involves coordination among multiple individuals. For example, related or unrelated individuals may care for offspring they did not produce (alloparental care) or group members in social carnivores may cooperate with one another to acquire food during group hunting.

We predicted that elevated GCs may motivate cooperative behavior in highly social species, such as cooperative breeders, regardless of whether the increase in GCs is caused by a social or environmental factor (Figure 2d). This is because highly social species may heavily rely on cooperation in coping with both social and nonsocial challenges and stressors. For example, a few studies in humans show that we act more cooperatively immediately after social stressors as well as in times of immediate emergency (Buchanan & Preston, 2014; von Dawans, Fischbacher, Kirschbaum, Fehr, & Heinrichs, 2012). However, long‐term cooperative strategies likely depend on the effectiveness of cooperation in ameliorating the stressors, and thus we expected that acute and chronic stress might have different effects on cooperative behavior. Research on the association between HPA‐axis activity and group‐level cooperation is rare, so we will primarily focus on the association between GCs and a specific type of cooperation: alloparental behavior (offspring guarding and provisioning) in cooperative breeders. In a cooperatively breeding group, alloparental behavior is carried out by subordinate “helpers” that stay in their natal group but rarely breed themselves, and instead help their parents breed, making alloparental behavior a good measure of *cooperative motivation*. However, as the effects of GCs on cooperative behavior likely reflect not only motivation to cooperate but also the mechanistic capacity to do so (Kasper et al., 2017), we supplement this discussion with evidence on the effects of GCs on *cooperative capability*, namely social coordination among group members in a cooperative context (Oliveira, Silva, & Canario, 2009).

#### 5.4.1 Evidence for the effect of GCs on alloparental behavior

**Figure 6:**
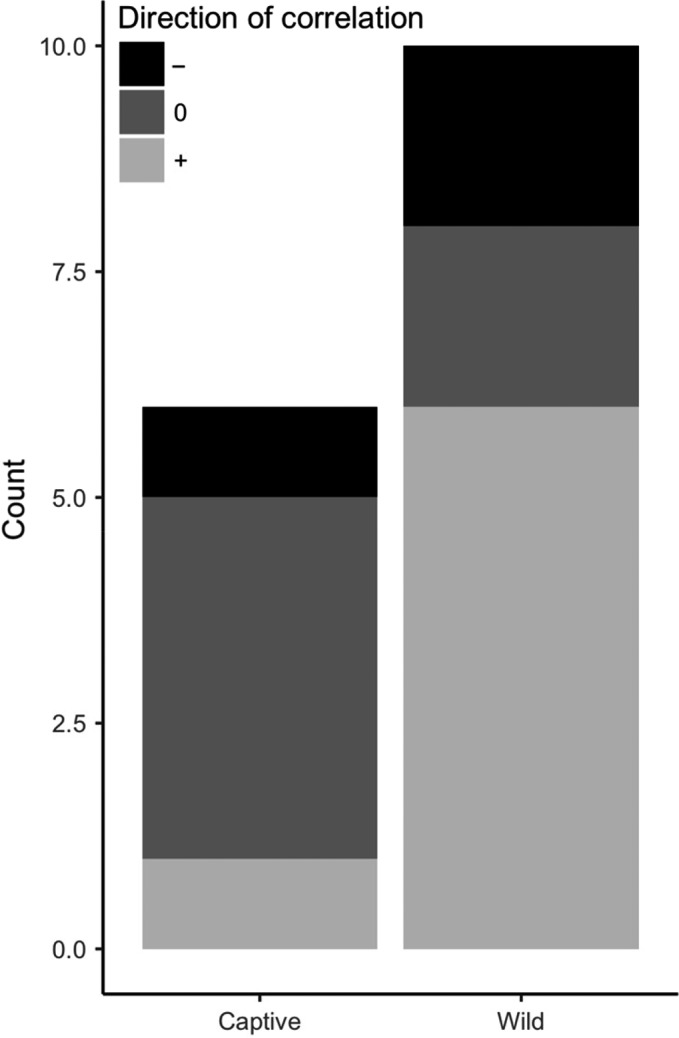
Summary of studies on the associations between measures of glucocorticoids (GCs) and alloparental behavior in wild and captive cooperative breeders. While most studies from captive studies found no correlation between alloparental behavior and GCs, the majority of the evidence from wild animals shows GCs are positively associated with alloparental behavior. Counts are comparisons where any measure of GCs and alloparental behavior were investigated and in some cases there was more than one comparison per study (see Table S4)

We found 12 studies in 7 cooperatively breeding species that measured associations between GCs and different types of alloparental care behavior (Table S4, Figure 6). Seven studies were done in wild populations and five in captive populations. Because of the small number of studies, we will review the evidence from both wild and captive animal studies together. We note that any causality needs to be inferred with care because cooperative behaviors can involve energetically‐demanding behaviors (such as feeding young) that can affect GC levels (e.g., Dantzer, Bennett, & Clutton‐Brock, 2017; Rivers, Newberry, Schwarz, & Ardia, 2017).

While a majority of the studies in wild and captive animals (60%) found some evidence supporting our prediction that individuals with higher GCs would exhibit more alloparental care, many of the comparisons of GCs and different types of alloparental behavior within these studies also found no significant association (Figure 6). There was some evidence that the effects of elevated HPA‐axis activity on alloparental care behavior was sex‐specific. For example, in both African striped mice (Raynaud & Schradin, 2015) and meerkats (Carlson, Manser, et al., 2006; Dantzer, Braga Goncalves, et al., 2017), elevated HPA‐axis activity increased alloparental behavior in males but not females, though in meerkats these effects depended upon the type of alloparental care behavior under consideration (Carlson, Russell, et al., 2006; Dantzer, Braga Goncalves, et al., 2017; Santema, Teitel, Manser, Bennett, & Clutton‐Brock, 2013). Two studies used an experimental approach in meerkats to show that elevated HPA‐axis activity in subordinate females decreased their expression of two types of alloparental care behaviors (Dantzer, Braga Goncalves, et al., 2017) but increased the amount of time they spent close to pups (Santema et al., 2013). Subordinate male meerkats with experimentally elevated HPA‐axis activity exhibited less pup‐guarding behavior (babysitting) but more food provisioning (pup feeding: Dantzer, Braga Goncalves, et al., 2017).

Although we found no studies examining how the duration of stress (acute vs. chronic) affected alloparental care, some studies showed how the timing of stress might be important for its effect on alloparenting. For example, in wild banded mongooses (*Mungos mungo*), males with higher pre‐breeding integrated GCs exhibited less alloparental care, but those with higher integrated GCs during the breeding period exhibited more alloparental care (*Mungos mungo*, Sanderson et al., 2014). Paralleling this, there was also some evidence that HPA‐axis activity can influence the probability that subordinates become or stay a helper in the first place and therefore have the opportunity to exhibit alloparental care. For example, in wild cooperatively breeding Superb Starlings (*Lamprotornis superbus*), individuals that had high pre‐breeding baseline GCs were more likely to become helpers (Rubenstein, 2007). During years of drought, these helpers had higher baseline and stress‐induced GCs during the breeding period and provided a larger proportion of nest‐provisioning than during years with more rainfall (Rubenstein, 2007). However, it is not yet clear if this decision to help is triggered by increased GCs caused by adverse weather (environmental stressor) or aggression from dominant breeders (social stressor), since adverse weather is also known to increase the amount of aggression dominant Superb Starlings direct at subordinates (Rubenstein, 2007).

#### 5.4.2 Evidence for the effect of GCs on social coordination among group members

In addition to their effects on alloparental care, GCs may also influence the capability to exhibit group‐level cooperative behavior by affecting social coordination among group members. Social coordination among group members consists of (i) event memory, (ii) synchrony with others, and (iii) social responsiveness to other group members (Kasper et al., 2017). An example of social coordination is cooperative hunting of large prey by social carnivores (e.g., canids or felids) where prey are pursued by group members. This cooperation requires coordinated behavior among group members (Bailey et al., 2013; Drea & Carter, 2009; Scheel & Packer, 1991). Hunters need to react similarly (have synchrony), communicate and pay attention to each other (have social responsiveness) and plan and predict what others are doing (have event memory) to be successful in the hunt. Individuals also need memory and responsiveness to others (assessment of social environment) when deciding with whom and when to cooperate (Soares et al., 2010).

Elevated HPA‐axis activity may have varied effects on these different aspects of social coordination among group members. First, GCs can affect event memory as different stressors are known to affect memory performance, both by impairing some aspects of memory while enhancing others (Packard et al., 2016; Takahashi et al., 2004; Zoladz et al., 2017). For instance, Zebra Finches with experimentally elevated GCs had lower event memory and were unable to sustain cooperation in a prisoner's dilemma task (Larose & Dubois, 2011).

Second, elevated HPA‐axis activity may promote synchrony among cooperating partners. For example, human stress responses in emergency situations may be highly contagious (Buchanan & Preston, 2014) and excitement may synchronize human brain activity (Nummenmaa et al., 2012). Furthermore, the stress of shared pain (e.g., eating a spicy chili in small groups) has been shown to enhance cooperation in humans (Bastian, Jetten, & Ferris, 2014). Endocrine synchrony in cooperative groups has not yet been studied, but evidence from comparable social settings suggests this possibility. For example, endocrine synchrony is associated with elevated parental cooperation (Hirschenhauser, Mostl, & Kotrschal, 1999; Ouyang, van Oers, Quetting, & Hau, 2014; Weiss, Kotrschal, Mostl, & Hirschenhauser, 2010). Physiologically synchronized individuals may exhibit less conflict with each other and may be more successful in conflicts with others (Soares et al., 2010).

Lastly, elevated HPA‐axis activity may also affect social coordination by affecting the responsiveness of individuals within the group to each other. Social responsiveness to other conspecifics (or empathy in humans) is a coordinating force behind a functioning group. It is needed both in maintaining social bonds or group stability and performing a coordinated cooperative behavior like group hunting. Even though the existence of actual empathy in nonhuman species is controversial, social responsiveness plays a key role in social behavior in all species inhabiting complex social groups. Because GCs may enhance vigilance and responsiveness to external stimuli in general, it is not surprising that empathy correlates positively with hormonal stress responsiveness in humans (Shirtcliff et al., 2009). This suggests the possibility that individuals with elevated HPA‐axis activity in cooperative species are more responsive to the state of other group members, potentially enhancing their cooperative capability.

#### 5.4.3 Discussion and future directions

We found evidence that increased HPA‐axis activity may promote cooperative behaviors among group members. This was limited to studies on the effects of GCs on alloparental care behavior and social coordination. Although the timing of the stressor may affect this relationship, we found no studies on whether the duration of stressor (acute vs. chronic) affects this relationship. Chronic elevations in GCs can reduce HPA‐axis responsiveness (Rich & Romero, 2005) and trigger strategies aimed at short‐term survival (Wingfield et al., 1998). Thus, when cooperation has only long‐term rewards, such as an increased chance of reproduction in the distant future, we predict that increased GCs are likely to reduce cooperative motivation and impair cooperative coordination. For example, after being experimentally stressed, female cleaner wrasses (*Labroides dimidiatus*) switched to a more selfish and less cooperative behavior where they bite nutritious mucus from their interspecific client fish partner (Soares, Cardoso, Grutter, Oliveira, & Bshary, 2014). This may be costly in terms of jeopardizing future partnerships with their clients, but provides more proximate benefits. On the other hand, chronic stress may also have opposite effects, tying social groups together. For example, exposure to chronic stress is known to tighten social networks, which results in cooperative or affiliative behavior limited more strictly to a group of close relatives or allies (humans: Dunbar & Spoors, 1995; Kornienko, Clemans, Out, & Granger, 2014; in other species: Zhou, Sornette, Hill, & Dunbar, 2005; baboons: Crockford et al., 2008; Wittig et al., 2008). Consequently, chronic stress may promote in‐group cooperation but also out‐group aggression (Puurtinen, Heap, & Mappes, 2014).

In highly interdependent cooperative species, cooperative behavior might be enhanced by environmental as well as social stress because cooperation among group members may be an effective (or in fact only) way to ameliorate stressful environments. For example, Savini, Boesch, and Reichard (2009) found that when inhabiting low quality home ranges, male gibbons (*Hylobates lar*) formed cooperative groups, whereas they usually are territorial in other areas. Similarly, an environmental stressor such as an increased threat of predation promotes cooperation (cooperative mobbing or brood care) in several bird species living in small groups (e.g., Pied Flycatcher: Krams et al., 2010; Common Eider, *Somateria mollissima*: Jaatinen, Ost, & Lehikoinen, 2011). This is in contrast to the noncooperative social species, such as colonially breeding sea birds, where environmental stressors that increase GCs mainly increase aggression and reduce social behavior (Common Guillemot, *Uria aalge*, Ashbrook, Wanless, Harris, & Hamer, 2008).

Taken together, these studies suggest that elevated HPA‐axis activity may motivate the expression of cooperative behavior, though the relationship is complex. Specifically, the effects of GCs on alloparental behavior seem to be sex‐specific and, as we suggested above, GCs might have different effects on cooperative behavior on short versus longer timescales. Future studies should investigate the interactive effects of GCs and other hormones on group‐level cooperative behavior. For example, Schoech, Mumme, and Wingfield (1996) found that individuals with higher prolactin levels showed more alloparental care behavior in cooperatively breeding Florida Scrub Jays (*Aphelocoma coerulescens*). Since GCs and prolactin may interact in motivating parental care under stress (see above), they may play an interactive role in alloparental care as well.

## 6 CONCLUSIONS

Our review suggests that the conventional negative relationship between HPA‐axis activity and social behavior is altered in more social species. The role of GCs in affecting the expression of social behavior appears to shift from *inhibitory* to more *facilitating* across a continuum of social behavior. Specifically, GCs seem to play an inhibitory role behind proactivity, be nonlinearly associated with parental care behavior, but show a facilitating role behind many forms of pair‐bonding affiliative behaviors and cooperation among group members (Figure 2). Across all categories of social behavior, severe or chronic elevation of GCs may eventually reduce social behavior, but the threshold of GCs before these consequences are reached may vary according to the context.

The *facilitating role* of GCs is evident because elaborate social behavior in pairs or groups may be an effective way to ameliorate the effects of stressors. Thus, the effects of GCs on social behavior may reflect the costs and benefits of sociality under adverse conditions. This balance of costs and benefits of sociality could be one key factor changing in the evolution of cooperative groups. Highly social species, where the presence of other group members can enhance individual survival or reproduction (at least for some group members), experience the highest benefits during adverse and stressful conditions, when they can allocate tasks among individuals and cooperation can elevate individual survival rates. On the other hand, less social species may experience the highest benefits during good conditions, when competition among group members is low.

We propose that the association between GCs and social behavior switches from inhibiting to facilitating during the evolution of highly social cooperative groups, mirroring the shifting balance of the costs and benefits of sociality. Following this, behavioral traits and endocrine systems might co‐evolve to achieve more synchronized stress responses and coordinated action among group members. Because group‐level cooperation is associated with out‐group competition, GCs might trigger increases in social behavior toward group members and aggression/avoidance toward other individuals at the same time (sensu De Dreu, 2012). We propose that this kind of group‐wide cooperative stress responses rise as follows:

1. Increased stress promotes (seeking) affiliation to reduce it;
2. Increased stress promotes (giving) affiliation to increase social support/bonding;
3. Increased stress in other individuals promotes affiliation to increase social support/bonding and to protect self from aggression;
4. Increased stress in others promotes concern for others and empathy and this promotes cooperative behavior;
5. Increased stress within the group promotes more or less synchronized stress response and cooperation among individuals as a way to adaptively respond to the stressor.

Overall, our review highlights the gap in our knowledge of how endocrine systems co‐vary with social behavioral profiles over evolutionary timescales where elevated HPA‐axis activity may reduce some types of social behavior (approaching conspecifics, parental care) but increase the expression of other types of social behavior found in group‐living species (pair‐bonding, alloparental care). These relationships are complex and may depend upon the environmental feature inducing the change in HPA‐axis activity (social vs. nonsocial factors) or the magnitude of the increase in HPA‐axis activity. We highlight specific areas of future research and provide a predictive framework (Figure 2) for future studies to increase our understanding of the mechanistic causes of individual variation in social behavior.

## CONFLICT OF INTEREST

None declared.

## AUTHOR CONTRIBUTIONS

A. Raulo did the literature analyses and plotted results. A. Raulo and B. Dantzer wrote and revised the manuscript.
